# Distinct Intrinsic and Extrinsic Factors Differentially Regulate Skeletal Stem Cells in Calvaria Versus Long Bones During Bone Regeneration

**DOI:** 10.3390/ijms26199413

**Published:** 2025-09-26

**Authors:** Jea Solidum, Kohei Yamasaki, Youngjae Jeong, Laura Ortinau, Francisco Heralde, Dongsu Park

**Affiliations:** 1 Department of Molecular Human Genetics, Baylor College of Medicine, One Baylor Plaza, Houston, TX 77030, USA; jnsolidum3@up.edu.ph (J.S.); youngjae.jeong@bcm.edu (Y.J.); laura.ortinau@bcm.edu (L.O.); 2 Department of Biochemistry and Molecular Biology, College of Medicine, University of the Philippines, Manila 1000, Philippines; 3 Center for Skeletal Biology, Baylor College of Medicine, One Baylor Plaza, Houston, TX 77030, USA

**Keywords:** suture stem cells, skeletal stem cells, cell transplantation, craniomaxillofacial, *Prx1*, chondrogenesis

## Abstract

Calvarial suture skeletal stem cells (Su-SSCs) are a distinct stem cell population for craniofacial bone formation by intramembranous ossification, compared to long bone periosteal SSCs (LB-PSSCs) with endochondral (osteochondrogenic) ossification. However, whether SSC intrinsic or extrinsic factors affect their differentiation process has not been well elucidated. Here, using an inducible *Prx1*-CreER-EGFP^+/−^;Rosa26-tdTomato mouse model, we observed that endogenous *Prx1*^+^ Su-SSCs and their orthotopic transplantation into calvarial injury do not form cartilage intermediates at the injury sites, while the transplantation of *Prx1*^+^ LB-PSSCs into LB injury induces osteochondrogenic differentiation, respectively. However, the heterotopic transplantation of *Prx1*^+^ Su-SSCs (Su-SSCs into LB injury) showed some surprising findings that the transplanted Su-SSCs acquire new chondrocyte differentiation properties at the LB injury sites, although the heterotopic-transplanted *Prx1*^+^ LB-PSSCs maintained their endochondral ossification properties at the calvarial injury sites. Further, a comparative single-cell transcriptomic analysis of LB-PSSCs and Su-SSCs revealed that Su-SSCs express a higher set of anti-chondrogenic genes, such as *Wnt5b*, *Twist1* while LB-PSSCs highly express chondrogenic *Hoxa-9*, *Hoxc-9*, *Hoxa-10*, *Hoxc-10,* and *Comp* genes. We also found that the heterotopic transplantation of LB-PSSCs into calvarial injury enhances bone healing in vivo. Taken together, these findings suggest that LB-PSSCs have high regenerative capability with invariable endochondral ossification even after the heterotopic transplantation but Su-SSCs are more flexible and regulated by the local bone environment. The transplantation of periosteal SSCs will be a promising method for large craniofacial bone defects.

## 1. Introduction

Congenital craniomaxillofacial (CMF) anomalies including malformations of the mouth, palate, face, and neck are one of the most clinically challenging and devastating defects across one’s lifespan. Additionally, traumatic injuries, cancers, or infectious diseases can cause CMF deformities, leading to many complications of patient life and care. While CMF surgery was ranked as the third most reconstructive procedure type performed in the United States [1], slow regeneration, complex structure, and severe pain of craniofacial bones still remain a significant issue in craniofacial reconstruction and tissue engineering.

Stem cell therapies for bone reconstruction have long been proposed. In craniofacial bones, calvarial sutures are the reservoir for suture SSCs (Su-SSCs), the main stem cell population for adult craniofacial bone regeneration and repair [2,3]. However, due to the difficulty in obtaining stem cells from craniofacial bones, the transplantation of Su-SSCs for CMF injuries is not feasible. Instead, bone marrow stromal cells (BMSCs) became a promising source for CMF reconstruction due to easy preparation and minimal side effects of donor sites [4,5,6,7]. However, previous studies showed that the structure and molecular regulation of long bone regeneration differs from those of craniofacial bones [8,9,10]. Further, it is well known that SSCs in long bones undergo endochondral ossification to develop bone and cartilage, while Su-SSCs undergo intramembranous ossification for craniofacial bone development [9]. Thus, long bones and craniofacial bones have distinct matrix structures and differences in their local tissue SSC microenvironmental factors and surrounding cells during the regeneration process [11,12,13,14]. In fact, how the different bone environments regulate endogenous and donor SSC function remains elusive.

We and others recently found that periosteal SSCs (PSSCs) are the main stem cells responsible for outer bone growth and cortical bone repair [15,16]. We also found LRP1+CD13+ P-SSCs with high osteochondrogenic potentials in human periosteum and their successful engraftment after transplantation into bone injuries of immunodeficient mice (NSG-EGFP) in vivo [17]. While recent studies reported the transcriptome and gene expression analysis with SSCs from different locations [17,18,19], detailed mechanisms to differentially regulate LB-PSSCs compared to those of Su-SSCs during regeneration and injury healing remain elusive. In particular, a major challenge of CMF regeneration is the difficulty in obtaining donor tissues and stem cells. Therefore, the application of other stem cells and their transplantation into CMF injuries is clinically relevant and an important question. However, whether LB-PSSC transplantation in CMF injuries can improve CMF bone healing and maintain their own differentiation properties after the transplantation or if they can acquire new intramembranous function is essentially unknown.

In this study, we sought to characterize how cell intrinsic and extrinsic factors regulate postnatal Su-SSCs and LB-PSSCs in the ossification process. Given that *Prx1*-CreER-GFP is a well-known inducible model for the labeling of postnatal periosteal and calvarial SSCs [20], we used *Prx1*-CreER-EGFP^+/−^;Rosa26-tdTomato mice for SSC (GFP^+^) isolation. To test whether SSC intrinsic or extrinsic factors can control their osteogenic and chondrogenic differentiation properties, we performed orthotopic transplantation (SSC transplantation into the same bone injury) of *Prx1*^+^ Su-SSCs and *Prx1*^+^ LB-PSSCs. Next, we performed heterotopic transplantation of *Prx1*^+^ LB-PSSCs to calvarial injury or *Prx1*^+^ Su-SSCs to tibial injury and sought to define whether Su-SSCs and LB-PSSCs have a differentiation plasticity. Additionally, using a comparative single-cell analysis, we identified a set of genes differentially expressed in *Prx1*^+^ LB-PSSCs and *Prx1*^+^ Su-SSCs, which potentially confer their pro-chondrogenic or anti-chondrogenic properties. This finding suggests a potential application and transplantation of the LB-PSSCs for other bone defect repair.

## 2. Results

### 2.1. Endogenous Prx1^+^ Su-SSCs Display Direct Osteogenic Differentiation (Intramembranous) After Bone Injury, While Prx1^+^ LB-PSSCs Undergo Endochondral Ossifications During Bone Healing

Postnatal SSCs are heterogeneous and LB-PSSCs are *Mx1*^+^ [21], Ctsk^+^ [22], and LRP1^+^CD13^+^ [17] cells with the expression of stem cell markers such as CD200 [23], while Su-SSCs are Gli1^+^ and Axin2^+^ cells [2,20,24]. However, there is no specific mouse model to compare LB-PSSCs with Su-SSCs in vivo.

**Figure 1:**
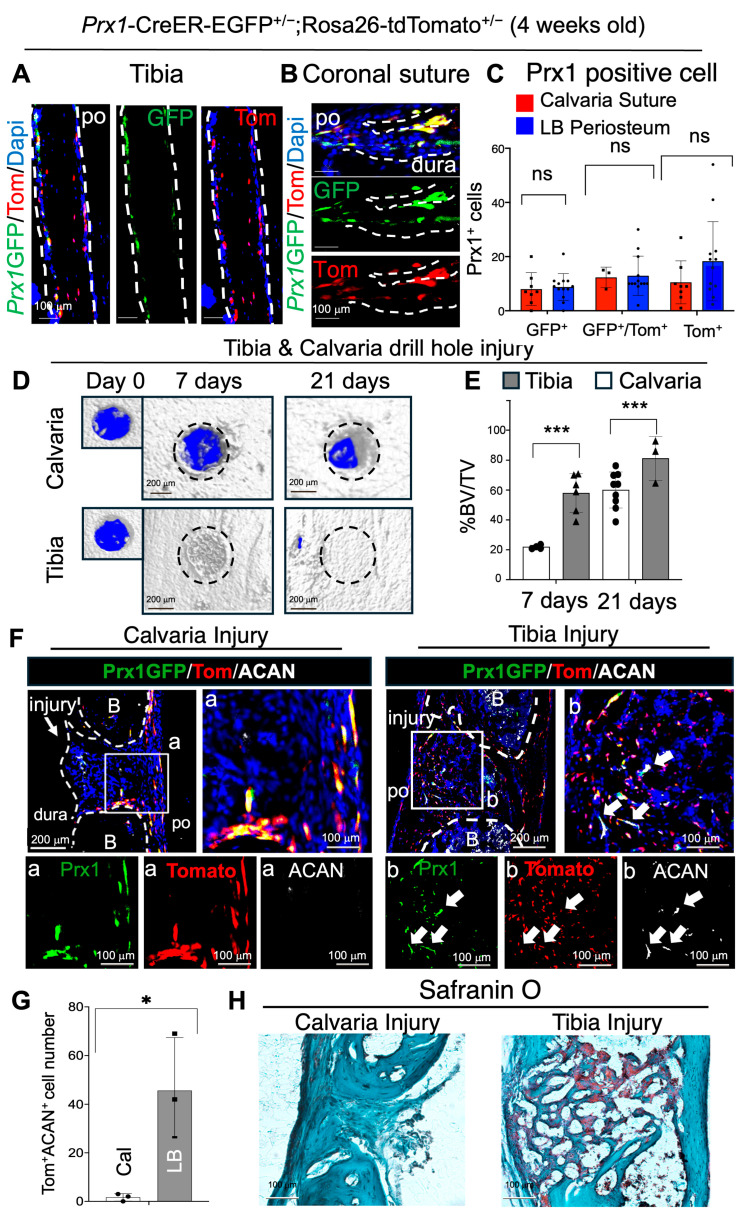
*Prx1*^+^ LB-PSSCs have higher injury responses with osteochondrogenic differentiation compared to *Prx1*^+^ Su-SSCs. (**A**,**B**) Immunofluorescence (IF) staining of 4-week tibial periosteum and calvarial sutures (**C**) and its quantification. White dotted line represents bone area. n.s: Not significant change (**D**) Micro-CT and (**E**) % BV/TV of calvarial and tibial injury. Dotted circle represent injury site. (**F**) IF staining of GFP, tomato, and aggrecan (ACAN) at the injury site of calvaria (21 days after injury) and tibia (7 days after the injury) and (**G**) the quantification of Tomato^+^ACAN^+^ cells. White arrows represent *Prx1* derived ACAN^+^ cell. White dotted line and letter B represents Bone. po: Periosteum. (**H**) Safranin O staining of calvarial and tibial injury. *: *p* < 0.05, ***: *p* < 0.001.

*Prx1* is a critical transcription factor for limb and craniofacial bone development. A previous study reported a *Prx1*-CreER-2A-EGFP model in which only *Prx1*^+^ skeletal stem/progenitor cells expressed GFP in long bone periosteum and calvarial sutures. However, when *Prx1*^+^ (GFP^+^) cells undergo differentiation, GFP is no longer expressed [20]. Although much is known about *Prx1*^+^ LB-PSSC function in LB development and bone healing, whether *Prx1*^+^ Su-SSCs possess osteochondrogenic potentials and how they contribute to calvarial bone healing is not clear. Therefore, to test and compare the function of *Prx1*^+^ Su-SSCs and *Prx1*^+^ LB-PSSCs in vivo, we first examined the number and location of *Prx1*^+^ SSCs (GFP^+^) and their descendant cells (GFP^−^Tomato^+^) in calvaria and tibia of 4-week-old *Prx1*-CreER-EGFP^+/−^;ROSA-tdTomato^+/−^ mice (Tamoxifen treatment at P8–P10). Consistent with the previous finding, we found that *Prx1*^+^ Su-SSCs were enriched in the calvarial sutures and *Prx1*^+^ LB-PSSCs were enriched in LB periosteum with varying but similar distribution (Figure 1A–C). In contrast, *Prx1*-labeled osteoprogenitor cells (GFP^+^Tomato^+^) and mature osteoblasts (GFP^−^Tomato^+^) were present in all bone compartments of calvarial and tibial bones, implying that the LB periosteum and calvarial sutures provide a specific microenvironment (niche) for endogenous SSC maintenance.

Next, to test osteochondrogenic potential and contribution of endogenous *Prx1*^+^ Su-SSCs and LB-PSSCs to the calvarial and tibial injury, respectively, we performed the same size (0.75 mm diameter) drill-hole defects in calvaria and tibia of 4-week-old *Prx1*-CreER-EGFP^+/−^;ROSA-tdTomato^+/−^ mice. Interestingly, subsequent µCT analysis showed that the healing of tibial defects was three times faster than those of calvarial defects at day 7, leading to near complete healing at 21 days post-injury. In contrast, calvarial defects remained ~50% healed at 21 days (Figure 1D,E). We also observed a greater number of *Prx1*-labeled cells (Tomato^+^) at the tibial injury site compared to calvarial injury. Next, to examine osteochondrogenic differentiation potential of *Prx1*^+^ SSCs in both injury sites, anti-aggrecan (ACAN, a chondrocyte marker) staining with tdTomato and GFP showed that a subset of *Prx1*-labeled cells (GFP^+^Tomato^+^ and/or Tomato^+^) in the tibia differentiated into ACAN^+^ chondrocytes, while those ACAN^+^ cells were not observed in the calvaria (Figure 1F,G). Further, Safranin O staining of calvarial and tibial injury sites showed that tibial injury, but not calvarial injury, formed cartilage intermediates during healing, as expected for endochondral ossification (Figure 1H). These results suggest that *Prx1*^+^ LB-PSSCs have greater proliferation and osteochondrogenic differentiation potential and contribute to better LB healing, compared to *Prx1*^+^ Su-SSCs.

### 2.2. Prx1^+^ LB-PSSCs Preserve Endochondral Ossification After Orthotopic and Heterotopic Transplantation

**Figure 2:**
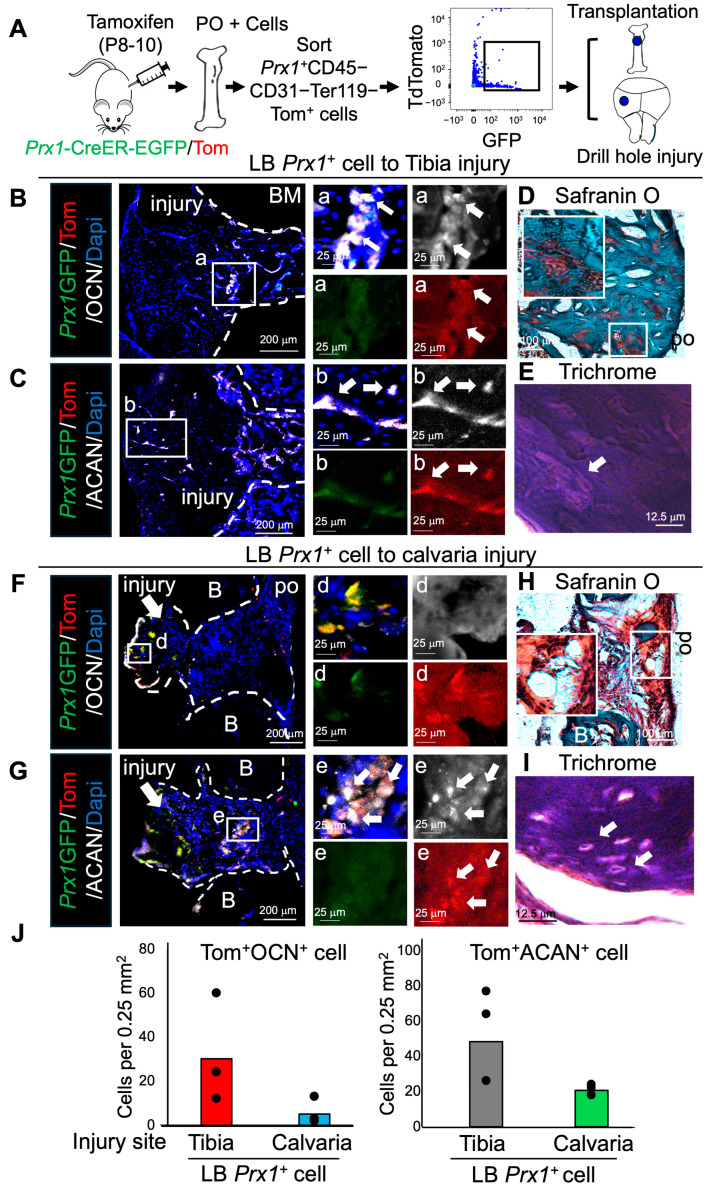
Osteochondrogenic injury healing of *Prx1*^+^ LB-PSSCs after orthotopic and heterotopic transplantation. (**A**) Experimental scheme for *Prx1*^+^ LB-PSSC isolation and transplantation into tibial (orthotopic) and calvarial (heterotopic) injuries. (**B**,**C**) IF staining of OCN and ACAN in the healing process of transplanted *Prx1*^+^ LB-PSSCs in tibial injury. White arrows represent *Prx1*^+^ cell derived chondrocyte and osteoblast. (**D**,**E**) Safranin O and Trichrome staining of tibial injury transplanted with *Prx1*^+^ LB-PSSCs. White arrow represents chondrocyte. (**F**,**G**) IF staining of OCN and ACAN in the healing process of transplanted *Prx1*^+^ LB-PSSCs in calvarial injury. White arrows represent *Prx1+* cell derived chondrocyte. (**H**,**I**) Safranin O and Trichrome staining. White arrow represents chondrocyte. (**J**) Quantification of Tomato^+^OCN^+^ and Tom^+^ACAN^+^ cells at the calvarial or tibial injury sites. White arrows in (**B**–**G**) indicate Tomato^+^OCN^+^ or Tomato^+^ACAN^+^ cells. n = 2–3 independent transplantation per group. B: Bone. po: Periosteum. BM: Bone marrow.

While bone grafts and stem cell transplantation are commonly used in orthopedic surgery, how niche factors in different bone environment affect the function of donor SSCs and their ossification process are not well defined. Therefore, we performed the transplantation of FACS-sorted *Prx1*^+^ LB-PSSCs and compared their differentiation in two different injury sites including tibial injury (orthotopic) and calvarial injury (heterotopic) (Figure 1A). To test their osteochondrogenic differentiation, we performed immunofluorescence (IF) staining of transplanted cells at the injury sites and found that a subset of *Prx1*^+^ LB-PSSCs differentiated toward chondrocytes (ACAN^+^) and osteoblasts (OCN^+^) in both calvarial and tibial injuries (Figure 2B,C,F,G,J). Further, safranin O and Trichrome staining showed that the transplantation of *Prx1*^+^ LB-PSSCs to both calvarial and tibial injuries led to endochondral ossification of healing (Figure 2D,E,H,I). These results suggest that *Prx1*^+^ LB-PSSC intrinsic factors retain PSSC proliferation and osteochondrogenic differentiation potentials, even after the heterotopic transplantation into different bone environment.

### 2.3. Prx1^+^ Su-SSCs Acquire Osteochondrogenic Differentiation Plasticity After Heterotopic Transplantation into Tibial Injury

**Figure 3:**
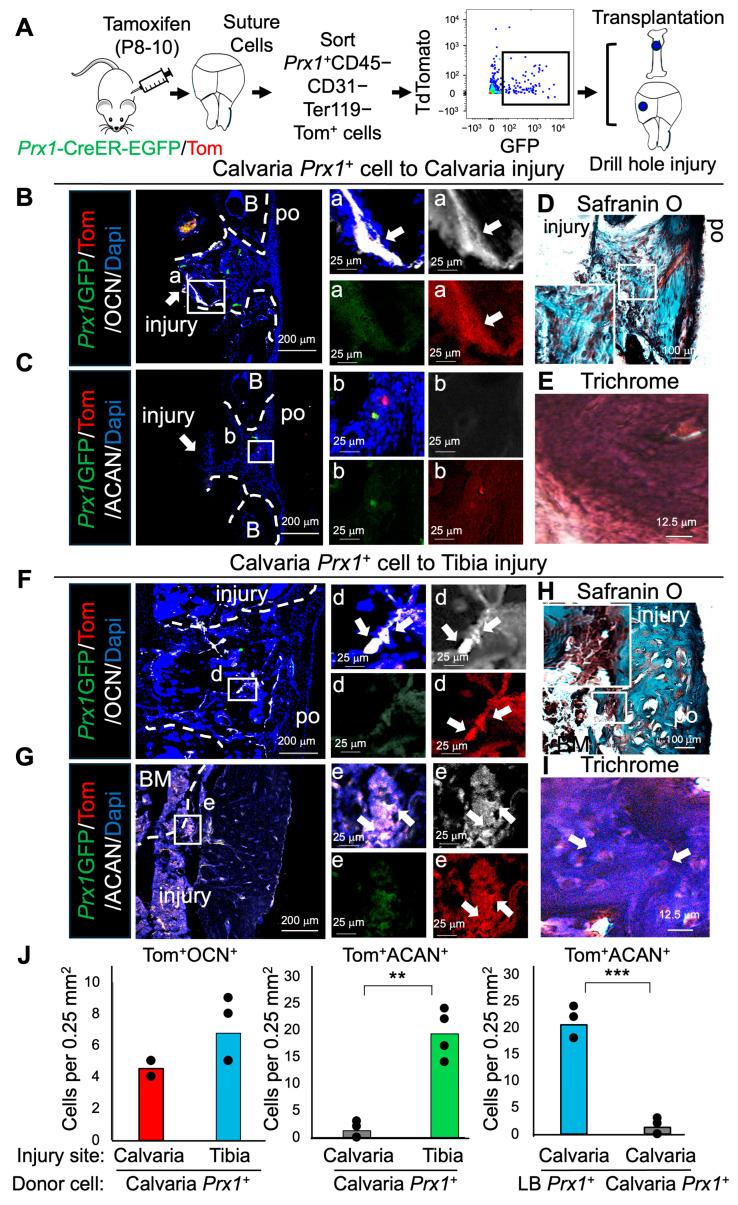
*Prx1*^+^ Su-SSCs are adaptable and their lineage differentiation can be controlled by environmental factors. (**A**) Experimental scheme for *Prx1*^+^ Su-SSC isolation and transplantation into calvarial (orthotopic) and tibial (heterotopic) injuries. (**B**,**C**) IF staining of OCN and ACAN in the healing process of transplanted *Prx1*^+^ Su-SSCs in calvarial injury. White arrows represent *Prx1+* cell derived osteoblasts. (**D**,**E**) Safranin O and Trichrome staining of calvarial injury transplanted with *Prx1*^+^ Su-SSCs. (**F**,**G**) IF staining of OCN and ACAN in the healing process of transplanted *Prx1*^+^ Su-SSCs in tibial injury White arrows represent *Prx1+* cell derived osteoblasts and chondrocytes. (**H**,**I**) Safranin O and Trichrome staining. White arrow represents chondrocyte. (**J**) Quantification of Tom^+^OCN^+^ cells and Tom^+^ACAN^+^ cells at the calvarial or tibial injury sites. **: *p* < 0.01, ***: *p* < 0.001. White arrow represents OCN or ACAN positive *Prx1*^+^ descendent cell. n = 2–3 independent transplantation per group. The images represent at least two independent staining. B: Bone. po: Periosteum. BM: Bone marrow.

Next, to test whether Su-SSCs preserve their intramembranous differentiation potentials, we used *Prx1*^+^ Su-SSCs as donor cells and transplanted them into calvarial (orthotopic) and tibial (heterotopic) injuries (Figure 3A). Immunostaining of engrafted *Prx1*^+^ Su-SSCs at the calvarial injury sites showed distinct differentiation toward Tomato^+^OCN^+^ osteogenic cells but undetectable Tomato^+^ACAN^+^ chondrogenic cells (Figure 3B,C). Safranin O and Trichrome staining further confirmed no positive staining within the Tomato^+^ cells, suggesting their intramembranous healing without formation of cartilage intermediates (Figure 3D,E). However, heterotopic transplantation of *Prx1*^+^ Su-SSCs to tibial injury led to unexpected differentiation of Su-SSCs toward Tomato^+^ACAN^+^ chondrogenic cells (Figure 3G,J) as well as Tomato^+^OCN^+^ osteogenic cells (Figure 3F,J). Safranin O and Trichrome staining also showed that there was also distinct cartilage formation (Figure 3H,I), supporting their endochondral ossification with the formation of cartilage intermediates. Taken together, these results suggest that *Prx1*^+^ Su-SSCs are adaptable and their lineage differentiation can be controlled by stem cell environmental factors. Specifically, the LB periosteal environment can stimulate the endochondral ossification of SSCs originating from different tissues.

### 2.4. Calvaria and Long Bones Have Distinct Sub-Cellular Clusters

**Figure 4:**
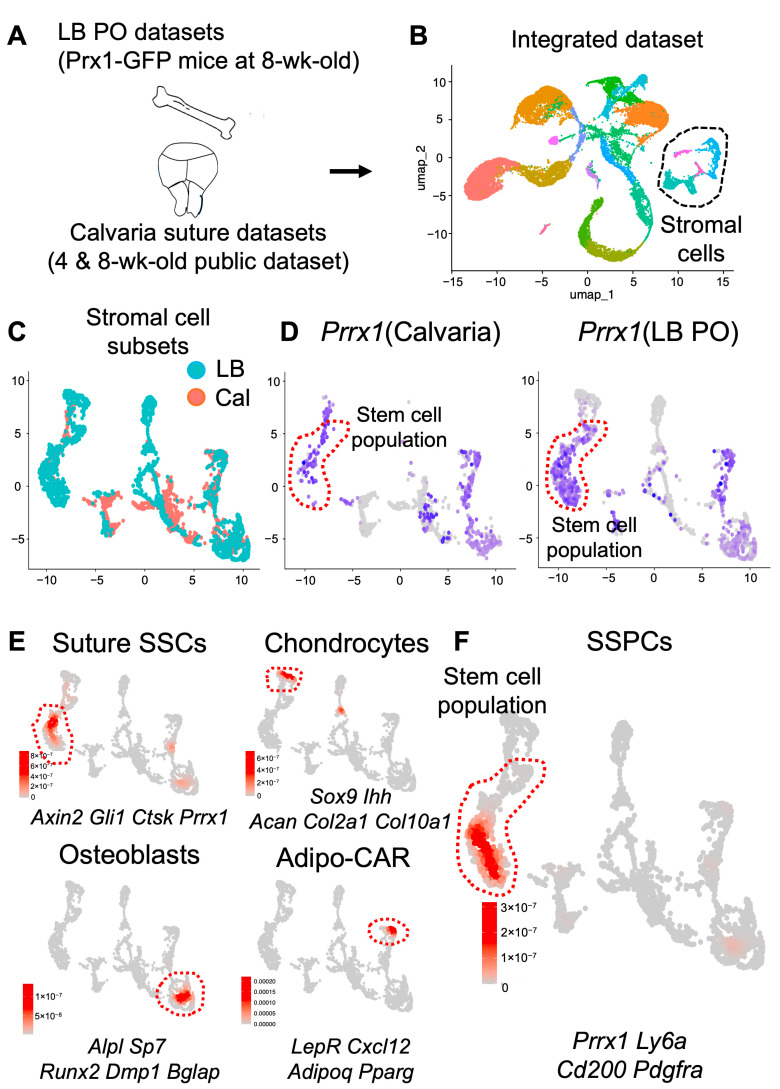
Comparative single-cell analysis of calvarial cells and long bone periosteal cells shows distinct sub-cellular *Prx1*^+^ clusters. (**A**) Schematic view of comparative single-cell data analysis using publicly available calvarial cell datasets and LB periosteal single-cell datasets from Prx1-GFP^+^ mice. (**B**) UMAP presentation of integrated datasets. Dot circle represents stromal cell clusters (**C**) Re-clustered UMAP analysis of stromal cells from LB periosteum (blue) and calvaria (red) single-cell datasets. (**D**) UMAP analysis of *Prrx1* expression in calvarial cells and long bone periosteal cell clusters. (**E**) Clusters (red circles) expressing BM/Adipo-CAR, suture SSCs, osteocyte, and chondrocyte markers listed in the bottom. (**F**) Stem cell population in subset stromal cell subsets. PO: Periosteum, BM: Bone marrow.

On the basis of these findings, we next defined subcellular compositions and gene expression of LB-PSSCs and Su-SSCs by performing a comparative single-cell transcriptomic analysis with our long bone periosteal datasets from 4-week-old *Prx1*-GFP^+^ mice [17] and previously reported datasets from 4- and 8-week-old mouse calvarial bones (Figure 4A). First, we assessed the integration of long bone (hind limbs) and calvarial sutures single-cell datasets (Figure 4B). Using uniform manifold approximation and projection (UMAP) clustering analysis and cell-type annotation analysis, we observed distinct stromal cell clusters according to differential gene expression and cell-type-specific markers including *Prx1* (P-SSC marker) (Figure 4B dot lines). We confirmed that other clusters including immune cells and endothelial cells highly overlapped in calvarial and long bone datasets (Figure 4B).

Subsequent integration of LB periosteal cells and calvarial stromal cells showed that there were several unique clusters in LB periosteal cells and calvarial stromal cells, although many clusters did overlap (Figure 4C). To further define which of these clusters are skeletal stem cells, we analyzed the transcript expression of common stem cell markers (i.e., *Prrx1*, *Ly6c*, *Cd200*, *Pdgfra*) and found that LB periosteal cells and calvarial stromal cells contained a common *Prx1*^+^ stem cell population (Figure 4D,F). Consistent with previous reports, we observed that osteogenic and adipogenic clusters between LB periosteal cells and calvarial stromal cells overlapped and shared similar gene expression. In contrast, osteochondroprogenitor and chondrogenic clusters were highly enriched in LB periosteal datasets but were not detectable in calvarial datasets, implying that long bones and calvarial bones have distinct progenitor clusters due to their different environmental regulation, but mature osteoblasts are similar in molecular level (Figure 4E).

### 2.5. Differential Expression of Pro-Chondrogenic Genes in Prx1^+^ LB-PSSCs and Anti-Chondrogenic Genes in Prx1^+^ Su-SSCs

**Figure 5:**
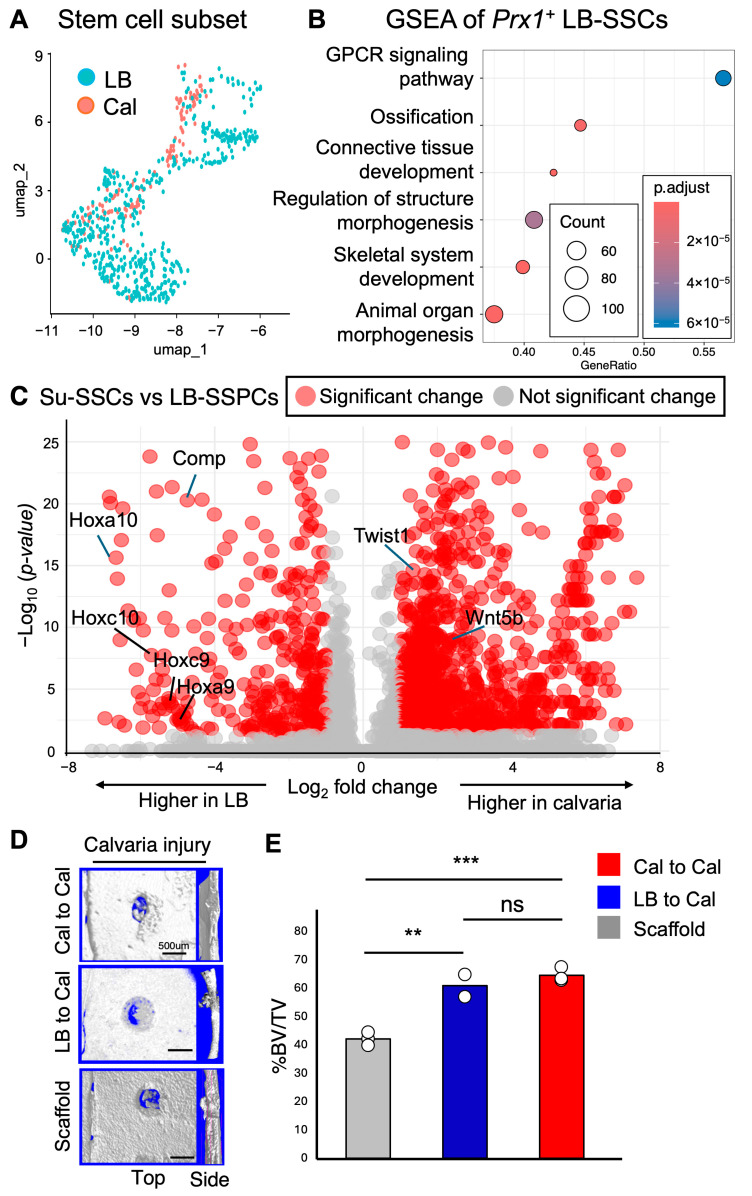
*Prx1*^+^ LB-PSSCs and *Prx1*^+^ Su-SSCs have distinct pro- and anti-chondrogenic gene expression properties. (**A**) Integrated UMAP analysis of stem cell clusters from LB and calvarial datasets. (**B**) Gene set enrichment analysis (GSEA) of *Prx1*^+^ LB-PSSCs compared with *Prx1*^+^ Su-SSCs. (**C**) Volcano plot analysis of differentially expressed genes (DEG) in *Prx1*^+^ Su-SSCs and LB-PSSCs. Genes of Log2FC < −1 and adjusted *p* value < 0.05 are represented as genes higher in LB. Genes of Log2FC > 1 and adjusted *p* value < 0.05 are represented as genes higher in calvaria. Cal: Calvaria. LB: Long bone. (**D**) Micro-CT analysis of calvarial injury healing after the transplantation of *Prx1*^+^ Su-SSCs or *Prx1*^+^ LB-PSSCs and (**E**) its quantification. n = 3 for calvarial stem cell transplantation for calvarial injury and scaffold control group. n = 2 for long bone stem cell transplantation for calvarial bone. **: *p* < 0.01, ***: *p* < 0.005 by Tukey’s test. n.s: not significant change.

To further define differentially expressed genes in LB-PSSCs and Su-SSCs, the *Prx1*^+^ stem cell clusters from long bone periosteal cells and calvarial stromal cells were separately grouped (Figure 5A) and used for gene set enrichment analysis (GSEA). Interestingly, we observed that *Prx1*^+^ LB-PSSCs were more heterogeneous with several subclusters compared to *Prx1*^+^ Su-SSCs (Figure 5A). Like other stem cells, comparative GSEA showed that *Prx1*^+^ LB-PSSCs highly expressed genes involved in bone development and cell morphogenesis. We also found that signaling pathways of G-protein coupled receptor were associated with *Prx1*^+^ LB-PSSCs. Consistent with their endochondral ossification potentials, *Prx1*^+^ LB-PSSCs highly expressed the transcripts related to cartilage and connective tissue development (Figure 5B). Next, differentially expressed gene (DEG) analysis between LB-SSCs and Su-SSCs revealed that *Prx1*^+^ LB-PSSCs had higher expression of *Hox* genes, specifically *Hoxa/c 9* and *Hoxa/c 10* genes critical for skeletal patterning and SSC differentiation to chondrocytes (Figure 5C) [25]. *Prx1*^+^ LB-PSSCs also highly expressed *Comp* gene (a known extracellular matrix gene to protect chondrocytes and cartilage) [26]. On the other hand, we observed that *Prx1*^+^ Su-SSCs highly expressed the anti-endochondral *Wnt5b* gene [27]. Additionally, they expressed *Twist1* (a gene associated with craniofacial abnormalities and craniosynostosis), supporting their intramembranous ossification property [28]. Together, comparative single-cell analysis of *Prx1*^+^LB-PSSCs and *Prx1*^+^ Su-SSCs and subsequent DEG analysis suggest that *Prx1*^+^ LB-PSSCs have higher pro-chondrogenic gene expression while *Prx1*^+^ Su-SSCs have high anti-chondrogenic properties.

### 2.6. Transplantation of Prx1^+^ LB-PSSCs Induces Comparable Healing of Calvarial Bone Injury

Given that slow healing, severe pain, and surgical complications remain major clinical challenges in traumatic craniofacial injuries, we next tested whether the heterotopic transplantation of LB-PSSCs can induce the healing of craniofacial bone injury in vivo. Calvarial injuries were induced in 6–8-week-old mice and *Prx1*^+^ LB-PSSCs and *Prx1*^+^ Su-SSCs (1 × 10^4^ cells in 5 µL of Matrigel) from 4-week-old *Prx1*-CreER-EGFP^+/−^;Rosa26-tdTomato mice (Tamoxifen at p8–p10) or Matrigel alone (as a control) were transplanted into calvarial injury sites on day 0. Subsequent µCT analysis of calvarial injury at day 14 showed a marked increase in new bone formation (BV/TV) when *Prx1*^+^ LB-PSSCs were transplanted at the sites of injury (Figure 5D,E). We also found that their bone-forming ability was similar to those of Su-SSCs. These results suggest that LB-PSSCs can be an alternative to SSCs that have comparable healing capability in calvarial bone injuries or critical bone defects in other bones.

## 3. Discussion

Tissue-resident SSCs are essential for the maintenance of adult bones that are morphologically and functionally variable, implying that SSCs from different bones (e.g., long bones vs. craniofacial bones) have distinct intrinsic and extrinsic regulatory mechanisms. However, how the different bone environment facilitates the function and regeneration of donor SSCs particularly after heterotopic transplantation (Su-SSCs into LB injury or LB-PSSCs into calvarial injury) remains unknown [29,30].

In this study, we showed that *Prx1*^+^ LB-PSSCs can differentiate toward chondrocytes (ACAN^+^) and osteoblasts (OCN^+^) in both orthotopic (tibial injury) and heterotopic (calvarial injury) transplantation. However, their proliferation and endochondral ossification capacity were greater in orthotopic transplantation compared to heterotopic transplantation, suggesting that LB-PSSC intrinsic factors maintain their endochondral ossification properties and LB niche factors also contribute to PSSC function (Figure 3H). Notably, *Prx1*^+^ Su-SSCs showed both chondrocyte and osteoblast differentiation when transplanted into tibial injury (heterotopic transplantation), while they only contributed to osteoblasts after the transplantation into calvarial injury (orthotopic transplantation). These results suggest that *Prx1*^+^ Su-SSCs have considerable plasticity, and extrinsic niche factors from bone environment can regulate their differentiation. Consistently, a previous study demonstrated that the fate of neural crest cells from mandibular periosteum can be altered in the tibia, implying the concept of cell plasticity [13]. While physical activity might differentially affect SSC function in different bones, our injury models were carefully controlled to ensure equivalent and minimal defect size (0.75 mm diameter) at both anatomical sites. Therefore, mechanical loading or physical activity likely did not contribute to these results. Taken together, our study suggests that both stem cell intrinsic and extrinsic factors potentially influence the type of SSC ossification and differentiation. Future studies will be required to define detailed molecular mechanisms and SSC niche factors.

Although we and others observed heterogeneity and functional difference of tissue-resident SSCs, the detail molecular features of SSCs in different bones are less well known. Our comparative scRNA-Seq analysis of LB-PSSCs and Su-SSCs showed higher expression of osteogenic and anti-chondrogenic genes in calvarial *Prx1*^+^ Su-SSC clusters while higher chondrogenic genes were expressed by *Prx1*^+^ LB-PSSC clusters. In addition, *Prx1*^+^ LB-PSSC clusters expressed higher levels of *Comp*, an ECM glycoprotein gene critical for ECM stability on chondrocytes and cartilage [26]. They also highly expressed gene transcripts responsible for stylopod patterning (*Hox9* and *Hox10* paralog) [31] and for articular chondrocyte formation (*Hand2* and *Gdf5*) [31,32]. On the other hand, *Prx1*^+^ Su-SSC clusters highly expressed gene transcripts related to anti-chondrogenesis, such as *Twist1* and *Wnt5b* [28]. *Wnt5b* was also reported to inhibit chondrocyte differentiation in the hind limb development [27]. Taken together, LB-PSSCs and Su-SSCs have distinct gene expression and cell intrinsic factors, which can lead *Prx1*^+^ Su-SSCs to intramembranous ossification and LB-PSSCs to endochondral ossification. While these findings are interesting, how *Prx1*^+^ Su-SSCs can obtain plasticity and differentiate into chondrocytes once they are transplanted in LB still remains unknown. Examining the gene expression changes of *Prx1*^+^ Su-SSCs after their transplantation into long bone injury sites is the next study to define which gene alterations can explain *Prx1*^+^ Su-SSC plasticity.

## 4. Materials and Methods

### 4.1. Animals

Six- to eight-week-old C57BL/6 (JAX:000664), *Prx1*-CreER-EGFP mice (JAX: 029211), and Rosa26LoxP-STOP-loxP-tdtomato mice (JAX: 007914) were purchased from The Jackson laboratory (Bar Harbor, ME, USA). Genotyping of these transgenic mice was performed according to The Jackson laboratory’s protocols. For *Prx1*-CreER recombinase induction, mice were injected intraperitoneally with 20 mg/kg of Tamoxifen dissolved in corn oil at P8–P10 or P53–55 for 4-week-old or 8-week-old mice experiments, respectively. All mice were maintained in pathogen-free conditions, and all procedures were approved as AN6721 by Institutional Animal Care and Use Committee of Baylor College of Medicine.

### 4.2. Histological Analysis

The calvaria or long bone tibia were dissected and fixed in 4% paraformaldehyde for 24–48 h at 4 °C followed by decalcification in 10% EDTA for 10–14 days. Decalcified tissue was placed in 10% sucrose solution overnight and moved to 30% sucrose solution for storage. The samples were embedded in O.C.T. Compound (Sakura Finetek USA, Inc., Torrance, CA, USA) before sectioning. The cryosection was performed by cryostat (CM3050S, Leica Biosystems, Nussloch, Germany) with the CryoJane tape transfer system (Leica Biosystems). For the sections labeled with fluorescent reporters, the slides were stained with DAPI and imaged by A1R-s confocal microscope (Nikon, Tokyo, Japan). The trichrome staining was performed using the trichrome stain kit (ab150686, Abcam, Cambridge, UK), according to the manufacturer’s instructions, and imaged by a Ci-L bright field microscope (Nikon).

### 4.3. Safranin O Histological Staining

Safranin O staining was performed using a general protocol to visualize cartilage formation and chondrocytes [22]. Briefly, Weigert’s hematoxylin was placed in slides with frozen sections for 5 min then rinsed with distilled water. The slides were dipped in 1% acid alcohol to wash and rinsed with distilled water. This was followed by 0.02% fast green staining for 1 min, 1% acetic acid for 30 s, and 0.1% Safranin O staining for 20 min, before rinsing briefly with 95% alcohol and dehydration with 100% alcohol. Sections were covered with cytoseal and coverslip before imaging. Cartilage was stained red in a background of green.

### 4.4. Immunofluorescence Staining

Frozen sections from long bones and calvarial bones fixed in 4% paraformaldehyde and decalcified were stained with goat or rabbit polyclonal anti-GFP antibody (ab5450, Abcam), rabbit polyclonal anti-Osteocalcin (ab93876, Abcam), and rabbit polyclonal anti-Aggrecan (ab216965, Abcam) according to the manufacturer’s instructions. Goat anti-rabbit Alexa Fluor 488 (A32731, Thermo Fisher Scientific, Waltham, MA, USA), and goat anti-rabbit Alexa Fluor 633 (A21070, Thermo Fisher Scientific) or donkey anti-rabbit 647 (A31573, Thermo Fisher Scientific) were used as secondary antibodies. Vectashield (Vector Labs, Newark, CA, USA) containing DAPI nuclear counterstain was used to mount the sections. Periosteal and suture structures were visualized by overlaying DAPI staining and polarized transmission lights. Images were acquired with an Eclipse Ti epifluorescence microscope (Nikon) equipped with a Q-imaging Micropublisher digital CCD color camera in the Optical Imaging and Vital Microscopy Core at Baylor College of Medicine. Images were processed with the NID-elements software (Version 4.60, Nikon Instruments).

### 4.5. Micro-CT Analysis

Tibial cortical bone and calvarial bone healing were assessed using Scanco μCT 40 (SCANCO Medical AG, Brüttisellen, Switzerland). Scans were performed with an X-ray setting of 55 kVp voltage and 145 µA current with a 200 ms exposure at a high resolution. Calibration images were collected prior to data acquisition. Scans were performed with an effective voxel size of 10 μm^3^. For image analysis, a global upper threshold of 255 and lower threshold of 110 (μCT grayscale value) were used for all samples to separate the bone from the background and soft tissue. CTan version 1.14.1 (Bruker, Billerica, MA, USA) was used to determine % bone volume over total volume.

### 4.6. Mouse Tibial and Calvarial Injury

All tibial and calvarial injuries were performed using aseptic technique on 1-month-old or 2-month-old male mice. For tibial injury, an incision less than 1 cm was made on the anterior side of the hind limb below the knee to visualize the proximal tibia. A 22G needle for a critical injury with 0.75 mm in diameter was used. The skin was closed using sutures and a small amount of triple antibiotic ointment was applied to the surrounding skin and sutures using a sterile cotton swab. For calvarial injury, a midline sagittal skin incision was made and a scalp flap was elevated to reveal the calvaria. The periosteum was retracted over the left and right parietal bones from the midsagittal incision to expose the underlying bone [2,33]. A 0.75 mm defect was made in the center of the left and right parietal bone. Injury healing was assessed 7 and 21 days post-injury.

### 4.7. Transplantation Experiment

*Postnatal Prx1*-GFP^+^ SSCs and *Prx1*-GFP^+^Tomato^+^ cells were isolated from the calvarial bones or long bone periosteum of 4-week-old *Prx1*-CreER-GFP^+/−^;tdTOMATO^+/−^ mice (Tamoxifen induced P8-10 donors). Bone defects in calvaria and tibia to 8-week-old C57BL/6 wild-type mice were made. Next, FACS-isolated *Prx1*-GFP^+^ SSCs and *Prx1*-GFP^+^Tomato^+^ cells (2500–10,000 cells/mouse) were mixed with Matrigel (5 µL)) and then transplanted into the tibial or calvarial defects. The new bone formation was assessed by µCT analysis at day 21 post-injury and transplantation. *Prx1*^+^ SSCs derived osteoblasts and cartilage chondrocyte were analyzed by anti-osteocalcin and anti-aggrecan staining with anti-GFP staining to amplify GFP signal intensity.

### 4.8. Single-Cell RNA Sequence Analysis

We used publicly available calvarial sagittal and lambdoid suture and long bone periosteum (hind limbs) single-cell datasets which were obtained from the NCBI Gene Expression Omnibus (GEO) and FaceBase Consortium under accession number GSE235176, FB00001199 and GSE276574. Raw read counts from the cells at each stage were analyzed using the Seurat package (version 5.2.1). Cells with more than 10% mitochondrial read content were filtered out following standard Seurat object generation. For the merged datasets, the SelectIntegrationFeatures function was performed before identifying anchors with the function FindIntegrationAnchors. Seurat objects were returned by passing these anchors to the IntegrateData function. Heterogeneity within the stromal cell populations was investigated through subcluster analysis. Published markers for the long bone, calvaria, and bone marrow were used to screen and identify the different cell populations.

### 4.9. Statistical Analysis

All measurements were made by a blinded examiner at 3 independent trials, and the average was recorded. All data were expressed as mean ± SEM or SD and individual data points. For comparison between 2 independent groups, statistical differences were evaluated by unpaired 2-tailed Student’s *t*-test. One-way analysis of variance, followed by Tukey’s post hoc test or Sidak correlation, was performed for multiple comparisons. A *p* value < 0.05 was considered statistically significant.

## 5. Conclusions

Craniofacial injury and reconstruction surgeries are often clinically challenging due to the limited source of donor bones and SSCs for transplantation. Our studies suggest that LB-PSSCs have a high regenerative capacity and maintain their ability to undergo endochondral ossification even after transplantation into long bones and craniofacial bones. Notably, our orthotopic transplantation of *Prx1*^+^ LB-PSSCs onto calvarial injury sites improved healing to a similar extent of *Prx1*^+^ Su-SSC transplantation. Thus, the manipulation and transplantation of PSSCs may lead to the development of new regenerative treatment methods for CMF and other bone injuries.

## Data Availability

All unique/stable reagents generated in this study are available from the lead contact with a completed Materials Transfer Agreement. Further information and requests for resources and reagents should be directed to and will be fulfilled by the lead contact, Dongsu Park (dongsu.park@bcm.edu).
